# Evaluation of the Relationship between Pain Exposure and Somatosensory Evoked Potentials in Preterm Infants: A Prospective Cohort Study

**DOI:** 10.3390/children11060676

**Published:** 2024-06-03

**Authors:** Caterina Coviello, Silvia Lori, Giovanna Bertini, Simona Montano, Simonetta Gabbanini, Maria Bastianelli, Cesarina Cossu, Sara Cavaliere, Clara Lunardi, Carlo Dani

**Affiliations:** 1 Division of Neonatology, Careggi University Hospital of Florence, 50134 Florence, Italy; giovanna.bertini@unifi.it (G.B.); simonamontano@libero.it (S.M.); clara.lunardi@fastwebnet.it (C.L.); carlo.dani@unifi.it (C.D.); 2 Neurophysiology Unit, Neuro-Musculo-Skeletal Department, Careggi University Hospital, 50134 Florence, Italy; silvia.lori@unifi.it (S.L.); gabbaninis@aou-careggi.toscana.it (S.G.); maria.bastianelli@unifi.it (M.B.); cossuc@aou-careggi.toscana.it (C.C.); cavalieres@aou-careggi.toscana.it (S.C.); 3 Department of Neurosciences, Psychology, Drug Research and Child Health, Careggi University Hospital of Florence, 50134 Florence, Italy

**Keywords:** somatosensory evoked potentials, pain, painful procedures, preterm infants

## Abstract

Background and aim: First, to compare somatosensory evoked potentials (SEPs) in preterm newborns without major brain injury studied at term equivalent age (TEA) with a term historical control group. Second, to investigate the impact of pain exposure during the first 28 days after birth on SEPs. Third, to evaluate the association between SEPs and Bayley-III at 2 years corrected age (CA). Methods: Infants born at <32 weeks’ gestational age (GA) were studied with continuous-SEPs. First, SEP differences between preterm and term infants were analyzed. Second, regression analyses were conducted to explore the association between SEPs and painful procedures, and then between SEPs and neurodevelopment. Results: 86 preterm infants were prospectively enrolled. Preterm infants exhibited prolonged N1 latencies, central conduction times (CCTs), lower N1-P1 amplitudes, and more recurrently abnormal SEPs compared to term infants. Higher pain exposure predicted longer N1 latency and slower CCT (all *p* < 0.005), adjusting for clinical risk factors. Younger GA and postmenstrual age (PMA) at SEP recording were associated with longer N1 latency and lower N1-P1 amplitude (all *p* < 0.005). A normal SEP at TEA positively predicted cognitive outcome at 2 years CA (*p* < 0.005). Conclusion: Pain exposure and prematurity were risk factors for altered SEP parameters at TEA. SEPs predicted cognitive outcome.

## 1. Introduction

Increased survival of preterm infants is a major success of current perinatal and neonatal medicine; however, these children may develop disabilities later in life, including neuromotor, cognitive, visual, and hearing impairments [1,2]. Behavioral and social problems are also described in follow-up studies [3,4]. Besides brain lesions [periventricular leukomalacia (PVL), intraventricular hemorrhage (IVH), cerebellar hemorrhage)], numerous risk factors have been recognized as responsible for unfavorable outcomes. Exposure to recurrent painful procedures during the neonatal period is one of those factors; in fact, the recent literature has demonstrated that early pain experiences may interfere with the regular growth of head circumference [5], with brain growth and maturation at term equivalent age (TEA) [6], and might have a negative impact on cognitive scores, neurosensory abilities, and psychosocial behaviors [7].

Cranial ultrasound scans and magnetic resonance imaging (MRI) are widely used in the neonatal period to identify brain injury and subsequent infants at risk of neurodevelopmental impairment [8,9,10]. In addition to neuroimaging, the study of somatosensory evoked potentials (SEPs) by electrical stimulation of the median or tibial nerve is a standard non-invasive neurophysiological test that evaluates the functional integrity of deep brain structures and cortical areas, giving information about the ability of the nervous system of a newborn to process external stimuli [11,12,13,14]. Cortical SEPs from median nerve stimulation can be recorded as early as 25 gestational weeks in infants [15]. After median nerve stimulation, the N1 response is the first contralateral parietal response in newborns, peaking about 30 ms after stimulation at TEA [13].

In addition, several reports have examined the prognostic role of SEPs for neurological outcomes in survivors from the neonatal intensive care unit (NICU) [16]. Abnormalities in median nerve SEPs have been associated with later cerebral palsy (CP), but with varying sensitivity and specificity [17,18,19].

The present study had three aims. First, to compare SEP waves and scores in a group of preterm newborns, without major brain injury studied at TEA with a term control group. Second, to investigate the impact of pain exposure on SEP waves and scores. Third, to evaluate the correlation between SEPs and neurodevelopmental outcome using the Bayley Scales of Infant and Toddler Development, third edition (Bayley-III) at 2 years corrected age (CA).

## 2. Methods

### 2.1. Study Population

Preterm neonates born <32 weeks GA, admitted to the NICU of the Careggi University Hospital of Florence between September 2018 and May 2021, were recruited for this prospective cohort study. The Pediatric Ethics Committee gave approval (112/2018), and informed written consent was obtained from all participants. Infants with congenital, genetic, and metabolic disorders and with severe brain injury, defined as intraventricular hemorrhage (IVH) ≥ 3 grade [20] and cystic periventricular leukomalacia (PVL) [21], were excluded.

The healthy infants cohort was a historical control group enrolled at the Careggi University Hospital between January 2013 and December 2013. Infants were born at term, with a normal weight for GA, and were less than 2 days old when they underwent multi-neurophysiological monitoring for research purposes [22].

### 2.2. Perinatal Data

Neonatal data were collected from daily chart reviews: GA, birth weight, type of delivery, gender, need and duration of mechanical ventilation, patent ductus arteriosus (PDA), occurrence of bronchopulmonary dysplasia (BPD) [23], postnatal steroids, culture-proven sepsis, necrotizing enterocolitis (NEC) [24], grade of IVH [20], and PVL [21]. Weight and head circumference z-scores at birth and at TEA were calculated according to the INES charts [25].

### 2.3. Pain Evaluation and Management

The exposure to neonatal invasive procedures was quantified by recording the number of skin-breaking procedures (heel lance, endotracheal intubation, peripheral intravenous or central line insertion, intra-muscular injection, chest tube and urinary catheter insertion, lumbar puncture) during the first 4 weeks after birth. For the following analyses, infants were grouped into low (lowest 50%) or high (highest 50%) painful procedures exposure, according to the median value.

In our NICU, pain management followed the Italian Society of Neonatology guidelines [26]. Newborns who underwent heel prick or peripheral line insertion received non-pharmacological analgesia. Before endotracheal intubation or central venous catheter insertion, an intravenous bolus of fentanyl (2 μg/kg) was administered. Infants on mechanical ventilation received fentanyl as their first choice, either as an intravenous bolus (1–3 μg/kg) or as a continuous infusion (0.5–3 μg/kg/h) when needed. In cases of prolonged ventilator dependence, morphine was introduced starting intravenously with a loading dose (0.05–0.1 mg/kg/dose), followed by a continuous infusion (0.01–0.05 mg/kg/h). Chest tube was positioned after an intravenous bolus of fentanyl (2–5 μg/kg) and a subcutaneous infiltration of lidocaine (up to 5 mg/kg). The Neonatal Pain Agitation and Sedation Scale was used for acute pain assessment [27].

### 2.4. Somatosensory Evoked Potential Recordings

Nemus-EB Neuro polygraph and GalNT/EP EXAM software (http://www.ebneuro.com/en/emg/nemus-1, 1 April 2024) were used for SEP recordings. Continuous SEPs were simultaneously obtained with video EEG (VEEG) from the same pool of electrodes, with the readings on-demand. The recording lasted nearly 1 h in order to obtain a complete spontaneous sleep cycle and awake phase. Since continuous SEPs were recorded contemporaneously with the VEEG cortical responses (N1 and P1) for both hemispheres, they were correlated to the behavioral states of active sleep, quiet sleep, and wakefulness in relation to the EEG [22]. The recordings were performed after feeding when the infants were quietly awake or asleep. Continuous SEP responses were elicited by electrical stimulation, alternating on both sides (Ag/AgCl disc electrodes) placed over the median nerves at the wrist bilaterally, applying a constant current square-wave pulse (rate 1.1 Hz). The stimulus time was 200 μs. The stimulus intensity was 10 to 20 mA and was augmented until it provoked a visible thumb twitch (motor threshold). SEPs were averaged over a 200 ms window (each average including 50 raw traces). The responses were recorded from the cortical (N1 and P1) and cervical (N13) levels and the reference electrode at Fz: C3’-Fz, C3’-C4’, C4’-Fz, C4’-C3’, and Cv7-Fz. The latency and amplitude markers of the main components (N1, P1, and N13) were labeled manually, thus the session was started. The software automatically placed the markers on the main components within a window of plus-minus 5 ms relative to the position of the markers on the previous trace. The traces of the continuous SEPs were displayed in cascade on modular screen windows, which showed the trend of the SEP recording time, intended as a progression that chronologically indicates latencies and amplitude. Only the cortical SEPs, with a clearly recognized normal cervical component (N13), were included in the study. Minimal and maximal values of cervical component responses were identified. For the left and right sides of N1, P1, and N13, mean values and standard deviations (SD) were calculated. Central conduction time (CCT) reflects the interpeak latency (IPL) between the N13 mean negative peak and N1 negative peak components [22]. The N1-P1 amplitude was also calculated. SEPs were also categorized by waveform morphology and the presence of modulation, following the score previously described by Lori et al. [28] as: bilateral normal responses (0), bilateral monotonous responses that were not modulated with respect to behavioral states (1), bilateral, reduced voltage-monotonous and/or increased latency responses (2), unilaterally absent responses (3), and bilaterally absent responses (4). For the analysis, the score was labeled as normal or abnormal.

### 2.5. Neurodevelopmental Outcome

The Bayley Scales of Infant and Toddler Development, third edition (BSITD-III), were applied to assess outcomes at 2 years CA. Cognitive and motor composite scores were standardized with a mean of 100 and a standard deviation of 15. 

### 2.6. Statistical Analysis

Statistical analysis was executed using IBM SPSS Statistics version 20. The infants’ clinical characteristics were described as mean and standard deviation, median and interquartile range (IQR), or rate and percentage. A paired sample *t*-test was performed to determine the difference between N1 latency, N1-P1 amplitude, and CCT of the left versus right side. Because no significant differences were found between the two sides (*p* > 0.05), the average values of the N1 latency, N1-P1 amplitude, and CCT were calculated for all subjects.

Our first aim was to compare SEP waves and scores between the preterm group and the term control group. Continuous variables were analyzed using the two-sample *t*-test, and categorical variables were analyzed using the Chi-Square test.

For the second aim, multivariate regression analyses were performed to explore the association between N1 latency, N1-P1 amplitude, and central conduction time (CCT) (as dependent variables) and painful procedures (high/low exposure), adjusting for GA, postmenstrual age (PMA) at recording, sepsis/NEC, and BPD. Sepsis and NEC were considered together since, in our population, all infants who suffered from NEC had a positive blood culture.

A linear regression analysis was then used to assess the association between the SEPs score (normal/abnormal) and painful procedures, adjusting for the previously mentioned comorbidities.

For the third aim, an additional multivariate regression analysis was performed to examine the correlation between the SEPs score and the Bayley-III scores at 2 years of CA, adjusting for GA at birth, painful procedures (high/low exposure), and BPD.

Results are expressed as coefficients of independent variables with 95% confidence intervals (CI).

## 3. Results

### 3.1. Subjects

Clinical data of the enrolled newborns are provided in Table 1.

The SEPs of 86 preterm infants were analyzed, all of good quality. SEPs were recorded at 39.3 ± 1.2 weeks of PMA. The median number of painful procedures during the first 4 weeks after birth was 55.

### 3.2. SEPs: Preterm vs. Term Infants

Preterm infants exhibited prolonged N1 latency, CCT, and lower amplitude compared to their pair term infants (*p* = 0.007, *p* = 0.002, and *p* = 0.016, respectively). In addition, a bilateral normal response was observed in 50% of preterm infants versus 100% of term infants (*p* = 0.001). No difference was observed in N13 latency (Table 2).

### 3.3. Assessment of Pain Exposure Affecting SEPs among Preterm Infants

**Figure 1:**
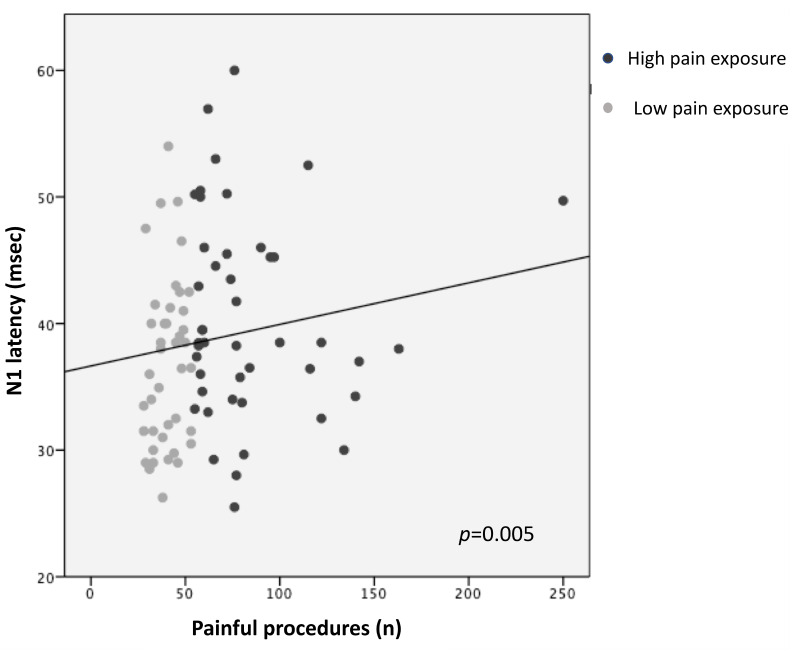
Correlation between N1 latency and pain exposure.

**Figure 2:**
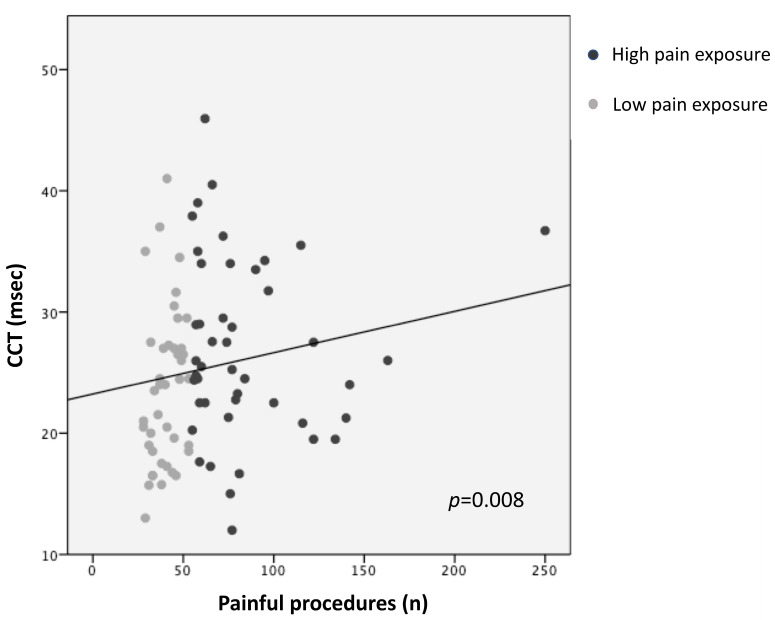
Correlation between central conduction time (CCT) and pain exposure.

Higher pain exposure predicted longer N1 latency and slower CCT (R^2^ = 0.14, *p* = 0.005; R^2^ = 0.12, *p* = 0.008, respectively), adjusting for GA, PMA at recording, sepsis/NEC, and BPD (Figure 1 and Figure 2; Table 3). Younger GA was associated with longer N1 latency (R^2^ = 0.14, *p* = 0.023) and lower N1-P1 amplitude (R^2^ = 0.13, *p* = 0.008), adjusting for PMA, sepsis/NEC, and BPD. PMA at recording was inversely correlated with N1 latency (R^2^ = 0.14, *p* = 0.048) and directly with N1-P1 amplitude (R^2^ = 0.13, *p* = 0.014) (Table 3).

Logistic regression analysis demonstrated that higher pain exposure predicted an increased risk of exhibiting an abnormal SEP at TEA, adjusting for GA, PMA at recording, sepsis/NEC, and BPD (Table 4).

### 3.4. SEPs and Outcome

Cognitive outcome at 2 years CA was positively predicted by the presence of a bilateral regular SEP (R^2^ =0.14, *p* = 0.004; Table 5), when adjusting for birth GA, BPD, and pain exposure. No correlation was found between SEPs score and motor outcome (Table 5).

## 4. Discussion

### 4.1. SEPs: Preterm vs. Term Infants

The first aim of our study was to compare SEP waves and scores in preterm infants evaluated at TEA with a healthy-term control group. We demonstrated that preterm infants, although without major brain injury, exhibited longer N1 latencies and CCTs, lower amplitudes, and more frequently abnormal SEPs compared to their pair born at term. Our results are supported by those of Smit [29], who reported that preterm infants presented greater N1 peak latencies compared to full-term babies, both at TEA and at 6 months CA [11,13,30,31]. These findings could suggest that extrauterine maturation of the somatosensory pathway in very preterm newborns might be delayed compared to the maturation in full-term infants [11,13,30,31], possibly because of a delay in central myelination but also for the immature functioning of the synapses [32,33]. Conversely, two other studies did not confirm our results; both studies included infants of older GA with a greater birth weight (GA < 37 weeks and birth weight > 1500 gr), compared to our cohort [31,34].

### 4.2. Assessment of Pain Exposure, GA, and PMA Affecting SEPs among Preterm Infants

We found that higher pain exposure during early life was associated with longer central latencies (N1 latency and CCT) and with an abnormal SEP at TEA. These relationships seemed to be independent from other comorbidities since they remained after adjusting for clinical risk factors. 

The last trimester of pregnancy, which corresponds to preterm birth, is a crucial period of brain development. During this epoch, the somatosensory processing pathway, which allows the transmission of tactile information, structurally develops and improves. Cortical Layer IV begins its differentiation between 20 and 26 weeks of gestation [35,36]. Thalamic afferents grow to the cortical plate and form the first thalamo-cortical connections, establishing the anatomical pathway for sensory impulses from the periphery to the cortex [37,38]. From 33 weeks, fibers from corpus callosum enter the cortex [39], dense intra-layer horizontal cortical projections progress by 37 weeks [35], and an intense axon progression increases within the parietal white matter at 38–42 weeks [40]. Myelination can be identified in the post-central gyri, where the primary somatosensory area is located, around the 35th GA [41]; it then continues its progression actively during the first postnatal year [42] and continues at a slower speed afterward.

Throughout such a critical phase, preterm infants experience hundreds of painful procedures during their hospital stay in order to prevent, diagnose, and treat critical conditions [43]. The nociceptive systems start to progress between 24 and 28 weeks of gestation, alongside the development of thalamocortical connections [44], while the descending endogenous modulation of the pain pathway only matures near the TEA [45]. Former reports have demonstrated that even a single skin-breaking procedure in preterm infants can be responsible for physiological, hormonal, inflammatory, electrophysiological responses, and hemodynamic modifications in the brain [46,47,48,49,50,51].

Furthermore, early exposure to pain has been found to interfere with brain maturation and later neurodevelopment in children born very preterm. In particular, recent studies reported that exposure to painful stimuli is associated with slower macrostructural growth of the thalami from early life to TEA [52], and consequently with smaller thalamic volumes at TEA, mainly localized to somatosensory regions [53]. In agreement with the volumetric findings, early pain exposure is responsible for slower thalamic metabolic growth, as indicated by a decreased N-acetylaspartate (NAA)/choline ratio in the thalami and microstructural alterations in thalamocortical pathways [53]. In addition, a recent study suggested that neonatal exposure to pain-related stress can induce alterations in the thalamocortical connectivity reflected by modifications in the spectral architecture of spontaneous brain oscillations [54].

SEP responses allow direct evaluation of the whole neural pathway from the skin through the thalamus to the cortex. These responses primarily depend on the development of thalamo-cortical connections in infants, with the N1 latency reflecting the functional integrity of the somatosensory pathway in the nervous system. In fact, in cases of damage to the thalamocortical projections, the N1 peak (latency) could be absent or delayed [29]. Thus, our findings of longer central latencies in more pain-exposed infants might be the reflection of an altered maturation or an injury of the thalami and/or of the thalamo-cortical connections.

Moreover, we observed that younger GA was also associated with longer N1 latency and lower amplitude. These findings are in line with our previous results, which showed that preterm infants exhibited longer latencies and smaller amplitudes at TEA compared with full-term newborns. Both of these results suggest that the maturation of the somatosensory system in preterm infants, even in the absence of severe brain injury, was delayed compared to that observed in full-term babies due to the interference of the extra uterine environment.

Furthermore, we found that PMA at recording was inversely correlated with N1 latency and directly with N1-P1 amplitude. Studies on preterm babies have shown that cortical SEPs can be documented since at least the 25th week of gestation [14,15]. In younger preterms, SEPs appear after moderately prolonged delays, and their waveforms typically exhibit small amplitude and long duration, possibly indicating a delayed myelination and immature synaptic connectivity of the nervous pathways [14,29,55]. With the progression of the maturation of the central and peripheral pathways, cortical latencies rapidly decrease, with the greatest variation observed between the near-term and term periods [12,14,30,34,56].

Interestingly, our findings indicated that higher pain exposure was correlated with an abnormal SEP. The SEPs score was determined by evaluating both waveform morphology and the presence of modulation. The modulation was assessed on the basis of trend-related SEPs related to behavioral states, since SEP responses were continuously and simultaneously recorded during VEEG. The presence of SEP modulation according to the brain state can offer a further indicator of brain integrity [57]. In fact, a previous study by Lori et al. found that the lack of modulation of continuous SEPs was associated with brain injury in term infants who suffered from perinatal asphyxia [28].

A regular cortical response at TEA is characterized by a sequence of positive and negative events, with a well-detectable N1 response approximately 30 ms after hand stimulation, reflecting the functional integrity of the somatosensory circuits and the maturation of the thalamo-cortical tracts [29].

Thus, this finding of abnormal SEPs correlated with pain exposure might also indicate that pain is responsible for a disruption of the somatosensory circuit.

To support this theory, existing evidence reports that children born preterm are at high risk for multiple sensorimotor problems, including difficulties with visual, auditory, tactile, balance, and kinesthetic processing [58,59,60,61], and exhibit more hyper- and hypo- sensory-sensitive behaviors compared to full-term-born children [62,63,64,65,66].

### 4.3. SEPs and Outcome

Lastly, we explored the relationship between the SEPs score and neurodevelopment. We found that cognitive outcome at 2 years CA was positively predicted by the presence of a regular SEP. On the contrary, we failed to find a correlation between SEPs and motor outcome. Earlier studies exploring the use of SEPs in the prediction of neurodevelopment in preterm subjects indicated that SEPs from the median nerve exhibit high sensitivity and specificity in predicting long-term neurodevelopmental outcomes. Three studies [16,17,67] studied the median nerve and demonstrated high prediction of CP. On the other side, the largest prospective study by De Vries et al. [13] showed less conclusive results. All these studies included infants with severe brain injuries [c-PVL, severe IVH (grade III or IV)] [16,17,67]. Our missing finding might reflect the characteristics of our cohort since none suffered from severe brain injury and only 9% of the infants showed low grade IVH.

The small sample size was one of the limitations of the present study, which might have restricted the possibility of detecting a significant role for other comorbidities. Second, regarding painful procedures, our research encountered the same critical issues as other studies in the field: we could only quantify the number of painful procedures, not the intensity of each painful stimulus.

## 5. Conclusions

Here, we demonstrated that preterm birth was correlated with an increased risk for altered somatosensory pathway maturation, even in the absence of severe brain injury. Early pain exposure, prematurity, and PMA affected SEP parameters at TEA. Pain exposure in preterm infants disrupts the development of the somatosensory system, and this in turn may have long-term effects on later development, indicating an enhanced vulnerability of the developing nervous system in younger GAs. An accurate and adequate management of pain in this high-risk population may aid in mitigating alterations in the somatosensory system, supporting extrauterine maturation and consequent neurodevelopment. In our cohort, SEPs at TEA were a good tool to predict cognitive outcomes.

## Figures and Tables

**Table 1:** Clinical characteristics of studied infants. Mean ± SD, median and (IQR), or rate and (%).

	*n* = 86
***Gestational age (wks)***	27.9 ± 1.9
***Male***	49 (57)
***Birth weight (gr)***	1012 ± 304
***Birth weight, Z score***	−0.08 ± 0.89
***Head circumference at birth (cm)***	25.2 ± 2.5
***Head circumference, z score at birth***	−0.16 ± 1.12
***Caesarean section***	62 (72)
***Apgar score at 5 min***	8 (8–8)
***Mechanical ventilation***	37 (43)
***Mechanical ventilation duration (d)***	5 (2–20)
***NICU stay (d)***	86 ± 29
***PMA at SEP recording (wks)***	39.3 ± 1.2
***Weight at SEP recording (g)***	2275 ± 377
***Weight z-score at SEP recording***	−2.14 ± 1.24
***Head circumference at SEP recording (cm)***	31.9 ± 1.6
***Head circumference z-score at SEP recording***	−1.7 ± 1.6
***PDA requiring treatment***	50 (58)
***NEC***	2 (2)
***BPD***	30 (35)
***Sepsis***	43 (50)
***IVH*** ***grade I*** ***grade II***	7 (8) 2 (2)
***Postnatal steroids***	22 (25)
***N° painful procedures 28 days***	55 (38–77)
***Cognitive outcome at 2 years***	92 ± 8
***Motor outcome at 2 years***	87 ± 6

PMA, post menstrual age; SEP, somatosensory evoked potentials; PDA, patent ductus arteriosus; NEC, necrotizing enterocolitis; BPD, bronchopulmonary dysplasia; IVH: intraventricular hemorrhage.

**Table 2:** SEP characteristics of preterm and term infants. Mean ± SD or rate and (%), two-sample *t*-test, and Chi-Square test.

	Preterm (*n* = 86)	Term (*n* = 20)	*p*
**Gestational age (wks) at SEP recording**	39.3 ± 1.2	39.8 ± 1.3	0.277
**N13 latency (ms)**	13.3 ± 0.23	13.6 ± 0.31	0.561
**N1 latency (ms)**	38.7 ± 0.8	33.9 ± 0.97	**0.007**
**CCT (ms)**	25.4 ± 0.7	20.3 ± 1.0	**0.002**
**N1-P1 amplitude (μV)**	3.2 ± 0.1	4.1 ± 0.5	**0.016**
**Score**			
**normal**			
0. bilateral normal responses	43 (50%)	20 (100%)	
**abnormal**			
1. bilateral monotonous responses that were not modulated with respect to behavioral states	32 (37%)	0 (0%)	**0.001**
2. bilateral, reduced voltage-monotonous and/or increased latency responses	10 (12%)	0 (0%)	
3. unilaterally absent responses	0 (0%)	0 (0%)	
4. bilaterally absent responses	1 (1%)	0 (0%)	

CCT, central conduction time.

**Table 3:** Multivariate linear regression analyses (backward elimination) between SEP parameters and clinical risk factors.

	N1 Latency (ms)			CCT (ms)			N1-P1 Amplitude (μV)		
	Adjusted R^2^ = 0.14			Adjusted R^2^ = 0.12			Adjusted R^2^ = 0.10		
	B	95% CI	*p*	B	95% CI	*p*	B	95%CI	*p*
**GA**	−0.061	−0.113–−0.008	**0.023**	0.008	−0.099–0.001	0.055	0.014	0.004–0.024	**0.008**
**PMA at recording**	−1.269	−2.528–−0.009	**0.048**	0.014	−2.254–0.156	0.087	0.302	0.064–0.540	**0.014**
**Sepsis/NEC**			-			-			-
**Painful procedures**	4.569	1.394–7.744	**0.005**	4.128	1.090–7.166	**0.008**			-
**BPD**			-			-			-

CCT, central conduction time; GA, gestational age; PMA, post menstrual age; NEC, necrotizing enterocolitis; BPD, bronchopulmonary dysplasia.

**Table 4:** Logistic regression analysis (backward elimination) between SEPs score and clinical risk factors.

	SEP Normal/Abnormal		
	R^2^ = 0.11		
	OR	95% CI	*p*
**GA**			-
**PMA at recording**			-
**Sepsis/NEC**			-
**Painful procedures**	3.434	1.406–8.386	**0.007**
**BPD**			-

SEP, somatosensory evoked potential; GA, gestational age; PMA, post menstrual age; NEC, necrotizing enterocolitis; BPD, bronchopulmonary dysplasia.

**Table 5:** Multivariate linear regression analysis (backward elimination) between SEPs score and neurological outcome at 2 years CA.

	Cognitive Outcome			Motor Outcome		
	Adjusted R^2^ = 0.14			Adjusted R^2^ = 0.11		
	B	95% CI	*p*	B	95% CI	*p*
**GA**	−1.442	−2.977–0.093	0.065			-
**BPD**			-			-
**Painful** **procedures**			-			-
**SEP** **normal/abnormal**	−8.685	−14.417–−2.954	**0.004**	−4.097	−8.700–0.507	0.079

GA, gestational age; BPD, bronchopulmonary dysplasia; SEP, somatosensory evoked potentials.

## Data Availability

All of the relevant data are within the manuscript.
